# Lavender (*Lavandula angustifolia* Mill.) Essential Oil Alleviates Neuropathic Pain in Mice With Spared Nerve Injury

**DOI:** 10.3389/fphar.2019.00472

**Published:** 2019-05-09

**Authors:** Maria Domenica Sanna, Francisco Les, Victor Lopez, Nicoletta Galeotti

**Affiliations:** ^1^ Section of Pharmacology, Department of Neuroscience, Psychology, Drug Research and Child Health (NEUROFARBA), University of Florence, Florence, Italy; ^2^ Department of Pharmacy, Faculty of Health Sciences, Universidad San Jorge, Zaragoza, Spain; ^3^ Instituto Agroalimentario de Aragón-IA2, CITA-Universidad de Zaragoza, Zaragoza, Spain

**Keywords:** lavender essential oil, central nervous system, neuropathic pain, anxiety, depression, memory, feeding

## Abstract

Low treatment efficacy represents an important unmet need in neuropathic pain patients and there is an urgent need to develop a more effective pharmacotherapy. An increasing number of patients choose complementary medicine to relieve pain. Lavender essential oil (LEO) is approved by the European Medicines Agency as herbal medicine to relieve anxiety and stress. However, the capability of LEO to relieve other nervous system disorders such as neuropathic pain has never been established. Our work aimed to evaluate the antineuropathic properties of lavender on a spared nerve injury (SNI) model of neuropathic pain in mice. An acute oral administration of LEO (100 mg/kg) alleviated SNI-induced mechanical allodynia, evaluated in the von Frey test, with an intensity comparable to the reference drug pregabalin. Investigations into the mechanism of action showed that LEO markedly decreased the phosphorylation of ERK1, ERK2, and JNK1, and decreased the levels of iNOS in the spinal cord; involvement of the endocannabinoid system was also detected using *in vitro* inhibition of the FAAH and MALG enzymes as well as *in vivo* experiments with the CB1 antagonist. Conversely, no effect on P38 phosphorylation and NF-kB activation was detected. These antihyperalgesic effects appeared at the same dose able to induce antidepressant-like, anxiolytic-like, and anorexic effects. In addition, gavage with LEO did not significantly alter animals’ gross behavior, motor coordination, or locomotor activity, nor impaired memory functions. Oral administration of LEO could represent a therapeutic approach in the management of neuropathic pain states.

## Introduction

Neuropathic pain involves a lesion or disease of the nervous system and comprises numerous chronic pain conditions that involve different pathophysiological mechanisms (Cohen and Mao, 2014). The neuropathic pain management is challenging. Despite the availability of numerous treatment options, many patients suffer from pain that is refractory to available treatments. Evaluation of pharmacotherapy in randomized clinical trials showed that clinically significant pain relief was experienced only by half of patients, which is predominantly partial but not complete relief. In addition, patients very often experience heavy side effects, and as a consequence, they discontinue therapy (Vranken, 2012; Finnerup et al., 2015). Inappropriate response to drug treatment represents an important unmet need in neuropathic pain patients. Therefore, there is an urgent need to develop novel and more effective therapies for this condition.

An increasing number of patients choose complementary or alternative medicine to relieve pain. Herbal medicines, commonly considered by population safer than conventional medicines, are widely used worldwide with more than 80% of the population consuming them for major and minor illnesses, particularly in developing countries (Ekor, 2014). Aromatherapy is one of the most widely used complementary therapies (Kyle, 2006); this discipline is based on the medicinal uses of essential oils extracted from aromatic plants, which can be absorbed through the skin (massage), the olfactory system (inhalation), or by oral administration (Buckle, 2014).

Lavender is one of the most popular essential oils used in aromatherapy. There are many different botanical species corresponding to the *Lavandula* genus but some of the species considered as medicinal are the following: *Lavandula angustifolia*, known as English lavender; *L. stoechas*, known as French lavender; *L. latifolia*, a Mediterranean grass-like lavender; and *L. intermedia*, which is a sterile cross between *L. latifolia* and *L. angustifolia*. The various lavenders have almost identical traditional uses and a large similarity in the main chemical constituents (Cavanagh and Wilkinson, 2002).

*L. angustifolia* Mill. is well known and appreciated by the cosmetic, food, and pharmaceutical industry as an aromatic and medicinal herb. Lavender essential oil is largely sold as over-the-counter herbal medicine for the treatment of depression, anxiety, and stress, and international organizations, including the World Health Organization (WHO), the European Medicines Agency (EMA), and the European Scientific Cooperative on Phytotherapy (ESCOP), approved lavender to ease anxiety, stress, and restlessness; a recent systematic review has also validated *L. angustifolia* essential oil for the treatment of generalized anxiety disorder (Barić et al., 2018).

Lavender is also used in many countries as complementary therapy for painful and inflammatory conditions (Djenane et al., 2012). Several clinical studies have reported that aromatherapy massage or inhalation with lavender produces pain relief in patients with different types of acute pain states, including pediatric pain (Soltani et al., 2013), dysmenorrhea (Ou et al., 2012), cesarean postoperative (Olapour et al., 2013), labor (Yazdkhasti and Pirak, 2016), and in inflammatory disorders, such as osteoarthritis (Nasiri et al., 2016). However, the capability of lavender essential oil to relieve neuropathic pain and its potential mechanisms has never been elucidated. This study aims to investigate the antineuropathic properties of lavender in a mouse model of neuropathic pain. Mood disorders, such as depression and anxiety, are frequently experienced by patients suffering from neuropathic pain (Langley et al., 2013). Epidemiologic studies reported approximately 34% mean prevalence rate for major depressive disorder in neuropathic patients (Gustorff et al., 2008) and clinical studies have indicated strong comorbidity between chronic pain and anxiety (Scott et al., 2007; Tsang et al., 2008). The induction of an antidepressant/anxiolytic action, along with an anti-allodynic activity, might improve the overall condition of patients and produce important clinical benefits. The capability of LEO to induce antidepressant/anxiolytic effects at analgesic doses is, thus, here investigated.

Finally, the induction of additional pharmacological or toxicological behavioral effects by the essential oil is also investigated in order to better define the lavender pharmacological and safety profile.

## Materials and Methods

### Animals

Experiments were performed on male CD1 mice (weight: 22–24 g; Harlan Laboratories, Bresso, Italy). Mice were randomly housed in standard cages and kept in a room at 23 ± 1°C with a 12-h light/dark cycle, light on at 7 a.m. Food (standard laboratory diet) and tap water were available *ad libitum*. The cages were placed in the experimental room 24 h before behavioral testing for acclimatization. All tests were conducted during the light phase. The experimental protocol was approved by the Institution’s Animal Care and Research Ethics Committee (University of Florence, Italy), under license from the Italian Department of Health (54/2014-B). Mice were treated in accordance with the relevant European Union (Directive 2010/63/EU, the council of 22 September 2010 on the protection of animals used for scientific purposes) and international regulations (Guide for the Care and Use of Laboratory Animals, US National Research Council, 2011). All studies involving animals are reported in accordance with the ARRIVE guidelines for experiments involving animals (McGrath and Lilley, 2015). The experimental protocol was designed to minimize the number of animals used and their suffering.

The number of animals per experiment was based on a power analysis (Charan and Kantharia, 2013). For behavioral assays, 10 animals per group were used to have the probability of 86% at which the study detects a difference between groups (0.05 significance level). A G power software was used to calculate sample size.

### Chemicals and Drug Administration

Pure lavender (*L. angustifolia*) essential oil (LEO) was kindly supplied by Pranarom International (Belgium). According to the GC-MS analyses performed by Pranarom, lavender essential oil (LEO) showed the following composition: linalyl acetate (35.96%), linalool (35.29%), β-caryophyllene (3.37%), trans-β-ocimene (2.88%), cis-β-ocimene (2.67%), lavandulyl acetate (2.29%), and terpinen-4-ol (1.94%).

Mice were randomly assigned to each treatment group by a researcher other than the operator. LEO was diluted in 5% DMSO and administered p.o. 30 min before testing at the dose of 100 mg/kg for all experiments except for dose-response curve where doses of LEO ranging from 10 to 200 mg/kg p.o. were used. The control group received equivalent volume of the vehicle.

Pregabalin (30 mg/kg i.p.) (Sigma, Milan, Italy) was administered 3 h before testing; citalopram hydrobromide (10 mg/kg i.p.), AM251 (4 mg/kg i.p.) (Tocris, Bristol, UK), diazepam (1 mg/kg i.p.), and amitriptyline (10 mg/kg i.p.) (Sigma, Milan, Italy) were administered 30 min before tests; morphine hydrochloride (7 mg/kg i.p.) (SALARS, Como, Italy) was administered 15 min before testing. The abovementioned reference drugs were dissolved in saline solution with the exception of AM251 that was prepared in a vehicle of dimethyl sulfoxide/Tween 80/0.9% saline (1:1:18). Treatments were administered in a volume of 10 ml/kg by gavage (p.o.) or intraperitoneal (i.p.) administration.

Behavioral tests on spared nerve injury (SNI) animals were performed on postoperative day 7. Samples to perform *in vitro* assays were harvested on postoperative day 7, 30 min after lavender oil administration.

### Nociceptive Behavior

#### Spared Nerve Injury

Behavioral testing was performed before surgery to establish a baseline for comparison with postsurgical values. The SNI procedure was applied as described by Bourquin et al. (2006). Mice were anesthetized (sodium pentobarbital 60 mg/kg i.p.) and placed in a prone position. An incision was made on the lateral surface of the thigh and a section was made directly through the biceps femoris muscle and the three branches of the sciatic nerve, the sural, common peroneal, and tibial nerves, were exposed. Both tibial and common peroneal nerves were ligated together (5.0 silk, Ethicon; Johnson & Johnson Intl, Brussels, Belgium) and transected approximately 2 mm distal to the ligation. The sural nerve was left intact. The muscles and skin were closed using a 5.0 silk suture. Sham-operated mice underwent the same procedure except ligation and transection of the nerves. Intense, reproducible, and long-lasting thermal and mechanical hypersensitivity is measurable in the non-injured sural nerve skin territory. Nociceptive threshold was measured on postsurgical day 7.

#### Mechanical Threshold (von Frey’s Test)

Mechanical allodynia was measured by using a Dynamic Plantar Aesthesiometer (Ugo Basile, Bologna, Italy) as previously described (Sanna et al., 2015). The mice were placed in individual Plexiglas chambers [8.5 × 3.4 × 3.4 (h) cm] on a wire-mesh grid floor and an adaptation period of approximately 1 h was allowed, during which exploratory and grooming activity ended. After that, a mechanical stimulus was delivered to the plantar surface of the hind paw of the mouse, using an automated transducer filament. A steel rod (2 mm) was pushed with electronic ascending force (0–5 g in 35 s). The pain threshold was defined as the amount of pressure, recorded to the nearest 0.1 g, that induced a flexor response and was determined by three repeated challenges at 10-min intervals. The averages of responses were calculated. Nociceptive response for mechanical sensitivity was expressed as paw withdrawal threshold (g) and both ipsilateral (operated) and contralateral (unoperated) hind paws were tested.

#### Hot Plate Test

The hot plate test was performed as previously described (Sanna et al., 2015). Mice were placed on a hot plate (Ugo Basile Biological Research Apparatus, Varese, Italy), with the temperature adjusted to 52.5 ± 0.1°C. Reaction latencies (s) were measured with a stopwatch before (baseline latency) and after treatments. The time to the first sign of nociception (paw licking) was recorded and the mouse immediately removed from the hot plate. An arbitrary cutoff period of 45s was adopted to avoid damage to the paws.

### Locomotor Activity

#### Rotarod Test

Motor performance was evaluated using the rotarod test (Galeotti et al., 2003). The rotarod apparatus consisted of a 3-cm-diameter rod with a non-slippery surface at a rotation rate of 16 RPM. The rod, 30 cm in length, was placed at a height of 15 cm from the base and divided into 5 equal sections by 6 disks. The animal was placed back on the rod immediately after falling and the integrity of motor coordination was assessed as number of falls from the rod in 30 s.

#### Hole-Board Test

The spontaneous locomotor activity was evaluated by using the hole-board test (Galeotti et al., 2003). The apparatus consisted of an elevated arena (40 cm × 40 cm; 1 m above the floor) with 16 evenly spaced holes (3 cm diameter; four lines of four holes each). Mice were placed individually on the center of the board and allowed to explore the plane freely for a period of 5 min each. Movements of the animal on the plane (spontaneous mobility) were automatically recorded by two photobeams, crossing the plane from midpoint to midpoint of opposite sides, thus dividing the plane into four equal quadrants. Miniature photoelectric cells, in each of the 16 holes, recorded the head-dips in the holes by the mice. This head-dipping behavior represents the exploratory activity of mice.

### Antidepressant-Like Activity

#### Tail Suspension Test

The tail suspension test was performed according to Galeotti and Ghelardini (2012). Mice were suspended from a plastic rod mounted 50 cm above the floor by adhesive tape placed to the upper middle of the tail. The time during which mice remained immobile was measured with a stopwatch during a test period of 6 min. Mice were considered immobile when they hung passively and completely motionless, except movements caused by respiration. Immobility was considered as depression-like behavior (behavioral despair) and was measured in the first 2 min of the test, when animals react to the unavoidable stress, and in the last 4 min, when the behavioral despair is established.

### Evaluation of Food Consumption

Feeding behavior was assessed in mice with no food provided for 4 h but water was available *ad libitum*. A weighed amount of food (standard laboratory pellets) was given to mice and the weight of the food consumed (difference between the given amount of food and that left in the cage, including spillage) was measured after 15, 30, 45, and 60 min, to an accuracy of 0.1 g.

### Evaluation of Mnemonic Functions

#### Novel Object Recognition Test

Memory-related responses were measured using the novel object recognition test (NORT), which is based on natural exploratory activity of mice. NORT evaluates the rodent’s ability to recognize a novel object in the environment and measures a form of recognition memory (Okamura et al., 2011). To perform the NORT, an open field device (cylinder diameter: 78 cm, height walls: 60 cm) was used. Mice were allowed to explore the open field. No object was placed in the box during the habituation session. Then, in the first session (training phase), animals were placed in the middle of the arena and presented with two identical objects (A1 and A2), placed 16 cm from the wall and 37 cm apart, for 5 min. Object exploration was measured manually using a stopwatch by an experienced observer blind to drug treatment. Exploration was defined as sniffing or touching the object with the nose or mouth. To measure short-term memory or long-term memory, the animals were placed back in the open field after 3-h or 24-h delay in the home cage, respectively, and presented with two objects, the familiar A1 (the same as the training phase) and a novel object B for 5 min (test phase). The objects were always placed in the same location. To secure the objects in place, Velcro into the base of the objects was used. Objects A1 and B had different shapes, colors, and sizes that had no significance for animals. The objects and the apparatus were cleaned with ethanol solution between trials to remove the olfactory cues. The test phase reflects the preference for the novel object. Recognition index for the novel object was calculated (TN-TF/TN + TF) × 100 (TF = time spent exploring familiar object; TN = time spent exploring the novel object). During the training session, both objects are novel and the time spent on both objects should be similar.

### Anxiolytic-Like Activity

#### Light-Dark Box

The light-dark box apparatus (length 50 cm, width 20.5 cm, and height 19 cm) consisted of two equal acrylic compartments, one dark (black) and one illuminated by a 60-W bulb lamp (white). A dark insert (with black walls and lid, nontransparent for visible light) was used to divide the arena into two equal parts. The two compartments communicated by a small door (10 cm × 3.2 cm) at floor level in the wall of the insert that allowed animals to move freely from one compartment to another. Each mouse was released in the center of the light compartment with its head facing away from the door and allowed to explore the arena for 5 min. Behavioral parameters recorded were the latency to the first step into the dark compartment, the time spent in the light chamber and the number of full-body transitions between chambers since previously described as a reflection of anxiety in this apparatus (Bourin and Hascoët, 2003). After testing, animals were removed from the light-dark box and returned to their home cage in colony room. After each test, the apparatus was cleaned with 70% ethanol to remove the olfactory cues and allowed to dry before the next subject was tested. This test exploited the conflict between the animal’s tendency to explore a new environment and its fear of bright light.

### Preparation of Membranes

Spinal cord samples from control and SNI mice were rapidly isolated and frozen in liquid nitrogen or stored at 80°C. The frozen spinal cords were homogenized in a lysis buffer. The homogenate was centrifuged at 9,000 × *g* for 15 min at 4°C, the low-speed pellet was discarded and the supernatant (total proteins) was stored at −80°C. Protein concentration was quantified using Bradford’s method (protein assay kit, Bio-Rad Laboratories, Milan, Italy).

### Western Blot Analysis

Membrane homogenates (20–50 μg) were separated on 10% sodium dodecyl sulfate-polyacrylamide gel electrophoresis (SDS-PAGE). The resolved proteins were transferred onto polyvinylidene difluoride (PVDF) membranes (120 min at 100 V) using standard procedures. Membranes were blocked in PBST (PBS containing 0.1% Tween) containing 5% nonfat dry milk for 120 min and then rinsed three times in Washing Buffer for 5 min each. Membranes were incubated overnight at 4°C with specific antibodies against ERK1/2 (1:1,000); ERK/1/2 phosphorylated on Thr202/Tyr204 **(**p-ERK1/2; 1:1,000); iNOS (1:250); Iκ-Bα (1:1,000) (Santa Cruz Biotechnology Inc, CA, USA); p38MAPK (1:500); p38MAPK phosphorylated onThr180/Tyr182 (p-p38 MAPK, 1:250); JNK (1:750); JNK phosphorylated on Thr183/Tyr185 (p-JNK, 1:750) (Cell Signalling Technology, MA, USA). The blots were rinsed thrice in PBS containing 0.1% Tween and incubated with goat anti-rabbit horseradish peroxidase-conjugated secondary antisera (1:10,000) and left for 1 h at room temperature. Blots were then extensively rinsed, followed by visualization using chemiluminescence detection system (Pierce, Milan, Italy) and signal intensity (pixels/mm^2^) quantified (ImageJ, NIH). Original blots are reported in Supplementary Figure 1. Exposition and developing time used was standardized for all the blots. For each sample, the signal intensity was normalized to that of ß-actin (Santa Cruz Biotechnology, CA, USA), used as loading control for protein expression, and the expression level of the proteins is an average of the densities per band area from each group.

### *In vitro* Inhibition of Fatty Acid Amide Hydrolase and Monoacylglycerol Lipase

The *in vitro* direct inhibition of the FAAH enzyme was measured by a fluorescence procedure using a Synergy H1 hybrid multimode reader (Biotek) and following manufacturer’s instructions of a commercial kit (Cayman, item no. 10005196). JZL 195 (CAS: 1210004-12-8), provided by Cayman, was used as reference inhibitor.

The inhibition of the MAGL enzyme was evaluated using the same Biotek reader by a colorimetric procedure following manufacturer’s instructions of a commercial kit (Cayman, item no. 705192) with the same reference inhibitor JZL 195. In both cases, LEO was dissolved in DMSO at various concentrations.

### Statistical Analysis

Data are expressed as the mean ± s.e.mean. For Western blotting assays, data are mean of four individual experiments conducted in duplicate. Statistical analysis was performed using Student’s *t*-test, one-way ANOVA (followed by Tukey’s test for *post hoc* comparison) or two-way ANOVA (followed by Bonferroni test for *post hoc* analysis). Mean differences with *p* < 0.05 were considered statistically significant. To conduct statistical analyses, the computer program GraphPad Prism version 5.0 (GraphPad Software Inc., San Diego, CA, USA) was used.

## Results

### Antinociceptive Activity of Lavender Essential Oil

**Figure 1:**
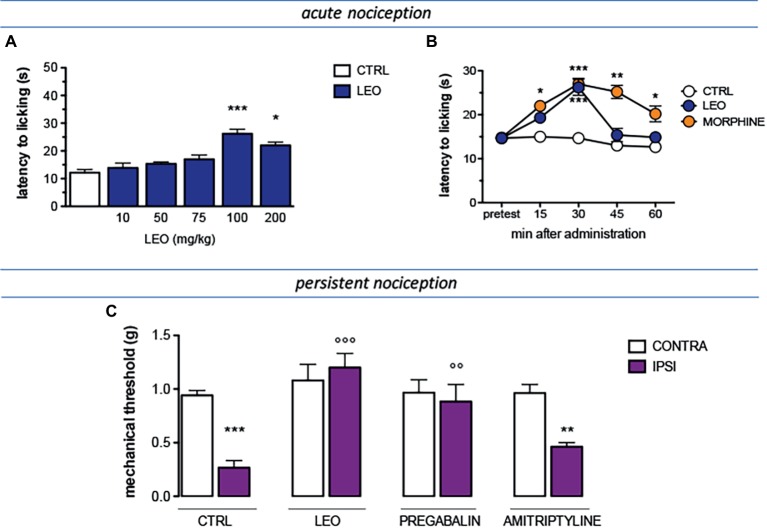
Antinociceptive profile of LEO. **(A)** A dose-response curve showed antinociceptive activity of LEO (10–200 mg/kg p.o.) against an acute thermal stimulus (hot plate test). (One-way ANOVA, *F*(5,59) = 23.51, *p* < 0.0001); *p* < 0.05; *p* < 0.001. **(B)** Time-course experiments with LEO (100 mg/kg p.o.) in comparison with morphine (7 mg/kg i.p.) in the hot plate test. (Two-way ANOVA, treatment *F*(2,135) = 41.08, *p* < 0.0001; time *F*(4,135) = 17.11, *p* < 0.001); *p < 0.05, **p < 0.01, ***p < 0.001 vs. control group. **(C)** Mice that underwent spared nerve injury (SNI) showed mechanical allodynia in the ipsilateral side in comparison with the contralateral side, on day 7 after surgery. LEO (100 mg/kg p.o.) prevented mechanical hypersensitivity. Pregabalin (30 mg/kg i.p.) and amitriptyline (10 mg/kg i.p.) were used as reference drugs. (One-way ANOVA, *F*(7,79) = 28.38, *p* < 0.0001); **p < 0.01, ***p < 0.001 vs. contralateral side; °°*p* < 0.01, °°°*p* < 0.001 vs. ipsilateral side.

The antinociceptive activity of lavender essential oil (LEO) was evaluated against conditions of acute and persistent nociception and the modulation of the pain threshold was evaluated by applying a thermal (hot plate test) or a mechanical (von Frey’s test) stimulus. A dose-response study showed that LEO produced a dose-related antinociceptive activity against an acute thermal stimulus. The doses of 10 and 50 mg/kg were ineffective. Although not significant, the dose of 75 mg/kg slightly increased the pain threshold whereas the dose of 100 mg/kg reached a significant antinociceptive activity. No further increase was detected at higher doses (Figure 1A). Time-course experiments showed that the antinociceptive effect of LEO 100 mg/kg peaked 30 min after administration and then rapidly diminished disappearing at 45 min (Figure 1B). The increase of pain threshold produced by LEO 15 and 30 min after administration was of intensity comparable to that produced by morphine 7 mg/kg s.c. (Figure 1B).

The antinociceptive activity of LEO was also investigated in the presence of a condition of persistent pain by using a model of neuropathic pain (SNI model) in mice. Seven days after surgery, SNI mice showed a marked mechanical hyperalgesia on the ipsilateral side in comparison with the contralateral uninjured side (Figure 1C). Treatment with LEO 100 mg/kg completely prevented mechanical allodynia in the ipsilateral side without any effect on the nociceptive threshold of the contralateral side with respect to values before treatment. The intensity of the antihyperalgesic effect produced by LEO was comparable to that induced by pregabalin, used as reference drug. Amitriptyline was used as negative reference drug and resulted ineffective (Figure 1C).

### Effect of Lavender Essential Oil on the Phosphorylation of MAPK

Large evidence indicates that MAPKs participate in the spinal mechanisms of neuropathic pain. Following LEO treatment, the levels of phosphorylation of ERK, JNK, and P38 within spinal cord of neuropathic mice were investigated in order to exploit the cellular pathway modulated by LEO in SNI animals.

**Figure 2:**
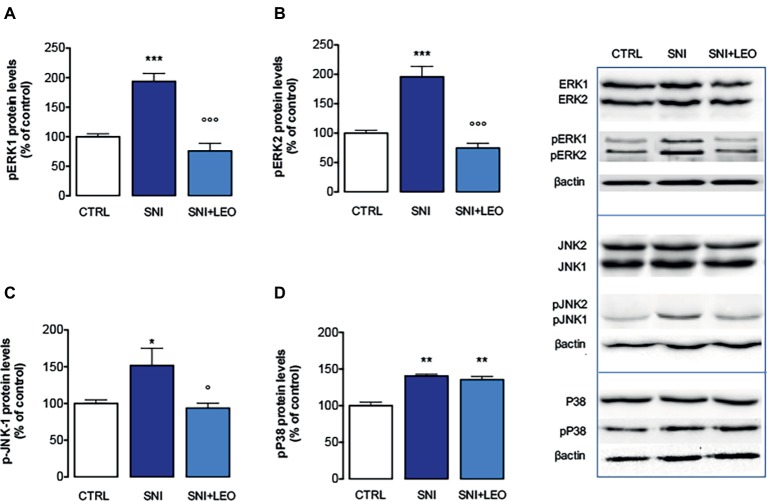
Effect of LEO on MAPK phosphorylation in the spinal cord of SNI mice. LEO (100 mg/kg p.o.) prevented the increase in the phosphorylation of ERK1 (one-way ANOVA, *F*(2,17) = 31.07, *p* < 0.0001) **(A)**, ERK2 (one-way ANOVA, *F*(2,17) = 30.81, *p* < 0.0001) **(B)**, and JNK1 (one-way ANOVA, *F*(2,17) = 9.80, *p* < 0.001) **(C)** induced by SNI 7 days after surgery. **(D)** Lack of effect of LEO on P38 increased phosphorylation (one-way ANOVA, *F*(2,17) = 23.73, *p* < 0.0001). Representative blots were reported in each panel. *p < 0.05, **p < 0.01, ***p < 0.001 vs. sham-operated control group. °*p* < 0.05, °°°*p* < 0.001 vs. SNI.

Experiments performed in spinal cord samples from the ipsilateral side of SNI mice, collected 7 days after surgery, showed an increase in the phosphorylation of both ERK1 (Figure 2A) and ERK2 (Figure 2B) isoforms. This ERK1 and ERK2 over-phosphorylation was prevented by treatment with LEO 100 mg/kg.

The analysis of levels of pJNK illustrated an isoform-selective influence of the SNI surgery procedure on activation of JNK proteins. Although JNK1 and JNK2 are abundantly expressed in the spinal cord, for the active forms, pJNK1 (p46) is the predominant spinal form and pJNK1 levels in the spinal cord selectively increase after nerve injury (Daulhac et al., 2006; Zhuang et al., 2006). In agreement with these studies, SNI mice showed increased levels of pJNK1. No band corresponding to pJNK2 molecular weight was detected. Treatment with LEO prevented the pJNK1 phosphorylation (Figures 2C,D).

An increase of p-P38 contents was observed in the spinal cord of SNI mice 7 days after surgery (Figure 2D). Converse to what was observed for ERK and JNK, treatment with LEO did not produce any variation in the levels of phosphorylated P38 MAPK (Figure 2D).

The expression of total ERK1/2, JNK1/2, and P38 was not significantly modified in spinal cord samples from SNI mice and no effect was produced by LEO administration (Figure 2).

### Effect of Lavender Essential Oil on the Activation of Inducible NO Synthase

**Figure 3:**
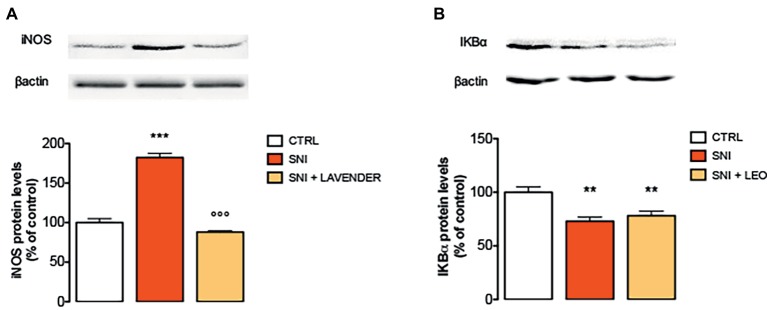
Effect of LEO on iNOS and NF-kB in the spinal cord of SNI mice. LEO (100 mg/kg p.o.) prevented the increased expression of iNOS **(A)** induced by SNI 7 days after surgery (One-way ANOVA, *F*(2,17) = 133.30, *p* < 0.0001). **(B)** Lack of effect of LEO on Ik-Balpha decreased expression (One-way ANOVA, *F*(2,17) = 12.27, *p* < 0.001). Representative blots were reported in each panel. **p < 0.01, ***p < 0.001 vs. sham-operated control group. °°°*p* < 0.001 vs. SNI.

Spinal neuroimmune and neuroinflammatory activities have been demonstrated to participate in neuropathic pain. Specifically, pro-inflammatory cytokines, such as IL-1, IL-6, TNF-α, and iNOS have been largely demonstrated to be involved in the onset and development of inflammatory and neuropathic pain (He et al., 2014). In the present study, SNI increased the expression of iNOS at day 7. Treatment with LEO 100 mg/kg significantly decreased the levels of iNOS compared to the SNI-treated group (Figure 3A).

### Lack of Effect of Lavender Essential Oil on the Activation of the NF-κB Pathway

To investigate transcriptional mechanisms that promote iNOS expression, the activation of NF-κB in SNI mice was examined by immunoblotting experiments. Under basal conditions, NF-κB is inhibited by the inhibitory subunit IκBα. Following phosphorylation and degradation of IκBα, NF-κB is released and translocates to the nucleus, promoting gene transcription. SNI mice showed a significant decrease of IκBα protein levels, indicating the degradation of the NF-κB inhibitory subunit, 7 days after surgery. However, LEO administration did not modify the IκBα levels indicating that it was unable to prevent the activation of the NF-κB pathway (Figure 3B).

### Involvement of the Endocannabinoid System: *In vitro* Inhibition of Fatty Acid Amide Hydrolase and Monoacylglycerol Lipase Enzymes and *in vivo* Pre-treatment With the CB1 Receptor Antagonist AM251

**Figure 4:**
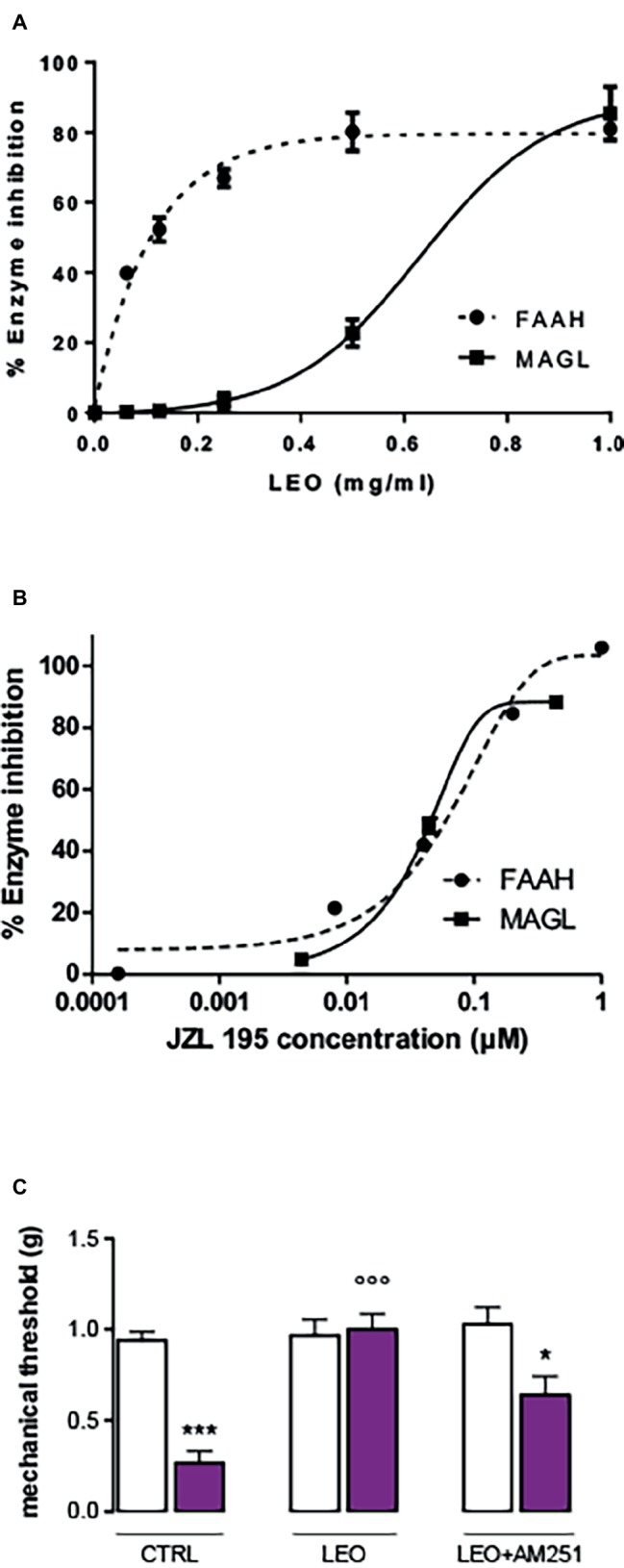
Inhibition of fatty acid amide hydrolase (FAAH) and monoacylglycerol lipase (MAGL) performed by lavender essential oil **(A)** and the reference inhibitor JZL 195 **(B)**. **(C)** Attenuation of the anti-allodynic effect FIGURE 4of LEO (100 mg/kg p.o.) by the CB1 receptor antagonist AM251 (4 mg/kg i.p.). (One-way ANOVA, *F*(5,59) = 24.29, *p* < 0.0001); *p < 0.05, ***p < 0.001 in comparison with CTRL contralateral side; °°°*p* < 0.001 in comparison with CTRL ipsilateral side.

In order to detect the potential implication of the endocannabinoid system in the analgesic activity of LEO, inhibition of the FAAH and MAGL enzymes was tested. LEO showed a dose-dependent response in the enzymatic bioassays (Figure 4A). The reference substance JZL 195 provided by Cayman showed a very similar profile both in the FAAH and MAGL enzymes with an IC50 value between 0.01 and 0.1 μM (Figure 4B). LEO was less potent than the inhibitor but was able to inhibit both enzymes, particularly FAAH (Figure 4A). Pre-treatment with the CB1 receptor antagonist AM251 (4 mg/kg) significantly attenuated LEO-induced anti-allodynic effect (Figure 4C).

### Antidepressant-Like Activity of Lavender Essential Oil in a Depressant-Like Paradigm

**Figure 5:**
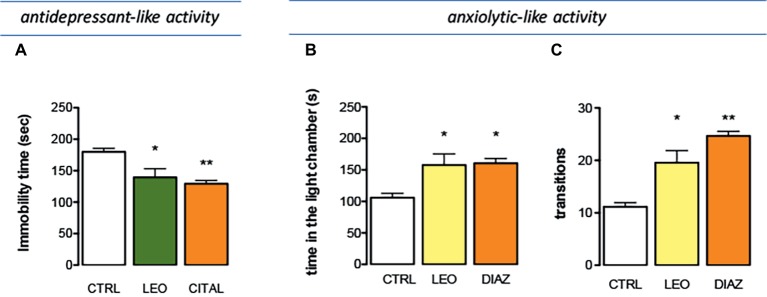
Effect of LEO on depression and anxiety. **(A)** Antidepressant-like effect induced by LEO (100 mg/kg p.o.) in the tail suspension test. Citalopram (CIT; 10 mg/kg i.p.) was used as antidepressant reference drug (one-way ANOVA, *F*(2,29) = 28.36, *p* < 0.0001). Anxiolytic-like activity of LEO showed by a reduction of the time spent in the light chamber (one-way ANOVA, *F*(2,29) = 14.42, *p* < 0.0001) **(B)** and by an increase in the number of transitions (one-way ANOVA, *F*(2,29) = 14.11, *p* < 0.0001) **(C)**. Diazepam (DIA; 1 mg/kg i.p.) was used as anxiolytic reference drug; *p < 0.05, **p < 0.001 in comparison with control mice.

The effect of LEO in a depressant-like behavior task was investigated in the tail suspension test (TST), one of the most widely used models for evaluating antidepressant-like activities in mice. Immobility developed by animals subjected to the short-term, unavoidable stress of being suspended by their tail is considered as a depressive-like behavior. The presence of an antidepressant-like phenotype is detected in the last 4 min of the test, when the behavioral despair is established. LEO decreased the immobility time values showing an antidepressant-like activity. The administration of citalopram, a reference antidepressant drug, reduced the immobility time values with an intensity comparable to that of LEO (Figure 5A).

### Anxiolytic-Like Activity of Lavender Essential Oil

The effect produced by LEO on anxiety-related behaviors was investigated by using the light-dark box test. Mice treated with LEO 100 mg/kg took less time to leave the dark compartment and spent significantly more time in the light chamber, thus evidencing anxiolytic-like properties. The anxiolytic compound diazepam was used as a positive reference drug. Diazepam prolonged the time spent in the lighted compartment producing an anxiolytic effect comparable to that induced by LEO (Figure 5B). The number of transitions between two boxes was a second behavioral parameter detected to evaluate the presence of an anxiolytic-like behavior. This parameter was significantly increased after LEO administration in comparison with the control group. This effect was comparable to that produced by diazepam (Figure 5C). All these data indicate an anxiolytic-like activity of LEO in mice.

### Anorexic Effect of Lavender Essential Oil

**Figure 6:**
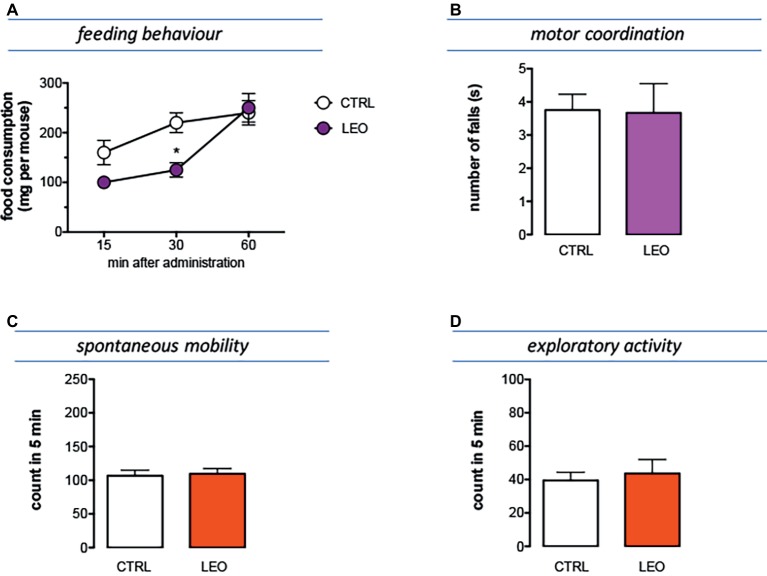
Effect of LEO on feeding and locomotor behaviour. **(A)** The food consumption was evaluated as the cumulated amount of food eaten over a 60-min period in 4 h food-deprived mice. LEO (100 mg/kg p.o.) significantly decreased food consumption 30 min after administration. (two-way ANOVA, treatment *F*(1,48) 16.72 *p* < 0.001; time *F*(2,48) 32.07 *p* < 0.0001); *p < 0.05 in comparison with control mice. Lack of impairment of motor coordination **(B)**, spontaneous mobility **(C)**, and exploratory activity **(D)** in mice treated with LEO.

The feeding behavior of mice treated with LEO 100 mg/kg was evaluated in animals that were previously deprived of food for 4 h, an experimental condition suitable to highlight both an increase and a decrease in food consumption. In Figure 5A is illustrated the cumulative curve for the food eaten by mice. Control mice showed a progressive increase of food intake in 60 min. Similarly, LEO-treated progressively increased the food consumption, but the cumulative amount of food eaten over the 60-min period of the test was significantly lower than control group, showing an anorexic phenotype (Figure 6A). The effect on feeding behavior disappeared 60 min after treatment, similar to what was observed for the antinociceptive activity.

### Lack of Impairment of Locomotor Behavior

Mice treated with LEO 100 mg/kg did not show any alteration in gross behavior or visible sign of poor health. In addition, specific tests were conducted to reveal locomotor alterations not visible to the operator. The rotarod test was used to evaluate motor coordination and the hole-board test to evaluate spontaneous mobility and exploratory activity of treated mice.

The number of falls from the rotating rod was comparable to that of control mice, indicating that LEO did not impair motor coordination (Figure 6B). In addition, the spontaneous mobility (Figure 6C) and exploratory activity (Figure 6D) were unaltered by LEO administration in comparison with the control mice.

### Lack of Effect of Lavender Essential Oil on Memory Functions

To investigate the effect of LEO treatment on memory processes, the behavioral response of treated mice in the novel object recognition test (NORT) was determined.

**Figure 7:**
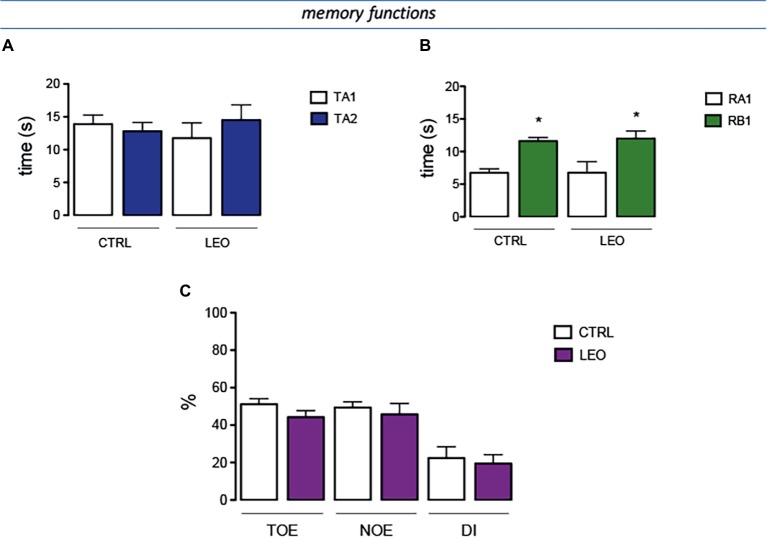
Lack of effect of LEO on memory processes. **(A)** In the training session of the novel object recognition test (NORT), no difference between exploration times was measured. **(B)** In the retention test, the exploration time of the familiar object was reduced in control group. LEO-treated mice (100 mg/kg p.o.) showed exploratory activity times similar to control mice. **(C)** LEO-treated mice showed a training object exploration index (TOE), novel object exploration index (NOE), and discrimination index (DI) comparable to control group. *p < 0.05 in comparison with training values (Student’s *t*-test).

In the training session of the NORT (internal control), the total time spent exploring both objects by LEO-treated mice was comparable to that of the control mice group (Figure 7A). Similarly, LEO-treated mice showed no differences with control group in exploration times (Figure 7A). In the retention session, the evaluation of the exploration times between training object and novel object illustrated that LEO treatment increased novel object exploration time with an intensity comparable to that detected in control mice (Figure 7B). The training object exploration index (TOE), the novel object exploration index (NOE), and the discrimination index (DI) of LEO-treated mice were similar to those of control mice, showing that LEO administration did not produce any detrimental or ameliorative effect on recognition memory (Figure 7C).

## Discussion

Pharmacological treatment is the mainstay of neuropathic pain management. Although there is a great number of available analgesic treatments, the unsatisfactory efficacy and negative side effects of these drugs make effective pharmacotherapy of neuropathic pain still often unobtainable (Finnerup et al., 2015). Thus, inadequate response to analgesic drugs still represents a major unmet need in the management of neuropathic pain. In order to exploit new therapeutic interventions for neuropathic pain, in the present study, the antihyperalgesic activity of lavender essential oil (LEO) was investigated in an animal model.

A single oral administration of LEO reversed mechanical hypersensitivity in the spared nerve injury (SNI) model of neuropathic pain, increasing the pain threshold to values comparable to that produced by pregabalin, one of the few drugs approved and licensed for neuropathic pain used as reference compound. Present results give the first description of the capability of LEO to ameliorate hyperalgesia in neuropathic pain conditions after a single oral administration. A recent clinical study reported the efficacy of topical application of the essential oil of *L. stoechas*, another plant from the genus *Lavandula*, on reducing pain intensity in mild to moderate carpal tunnel syndrome, the most common entrapment neuropathy of the upper extremity (Eftekharsadat et al., 2017), further supporting the hypothesis of a positive effect of lavender essential oils in neuropathic pain conditions.

Several clinical evaluations showed analgesic efficacy of aromatherapy massage or inhalation with LEO in different types of acute pain (Ou et al., 2012; Olapour et al., 2013; Soltani et al., 2013; Yazdkhasti and Pirak, 2016). To better define the antinociceptive profile of LEO, we also tested the essential oil in a condition of acute thermal pain. In the hot plate test, LEO increased the pain threshold with an efficacy similar to that produced by morphine (7 mg/kg), in agreement with clinical data. Furthermore, the antinociceptive effect was produced after oral administration, indicating that the analgesic properties of LEO were not strictly related to aromatherapy massage or inhalation of the essential oil. These results show that orally administered LEO is endowed with both antihyperalgesic and analgesic properties.

MAPK is a family of proteins composed by three major members: extracellular signal-regulated kinases (ERK), P38, and c-Jun N-terminal kinase (JNK), which represent three separate signaling pathways. A large evidence shows that activation of MAPK signaling has a critical role in peripheral and central sensitization associated with neuropathic pain conditions (Ji et al., 2009; Edelmayer et al., 2014). To evaluate the mechanism of antinociceptive action of LEO in neuropathic pain, the role of MAPK was investigated in spinal cord preparations from SNI mice. As previously reported, SNI increases phosphorylation of all MAPK members, even if with a different pattern of activation (Sanna et al., 2015). However, spinal ERK1, ERK2, P38, and JNK1, the predominant JNK active form in the spinal cord after nerve injury (Daulhac et al., 2006; Zhuang et al., 2006), were all over-phosphorylated 7 days after injury. Oral administration of LEO prevented the increase of ERK1, ERK2, and JNK1 phosphorylation. Conversely, no reduction in the levels of pP38 was detected. These findings indicate a prominent modulation of spinal ERK and JNK activity by LEO, whereas P38 appears not to be a key site of action for the LEO-induced anti-allodynic effect.

Recently, a growing number of literature reports have described the role of neuroinflammation in the pathogenesis of neuropathic pain (Austin and Moalem Taylor, 2010; Calvo et al., 2012; He et al., 2014). Pro-inflammatory cytokines such as IL-1, IL-6, TNF-α, and nitric oxide (NO) have been involved in demyelination and degeneration of peripheral nerves, increase in excitability of sensory afferent, and strongly implicated in the initiation and development of neuropathic pain (Jancálek et al., 2010; Ahlawat et al., 2014; He et al., 2014).

NO has been reported to participate in pain transmission. In addition, neuronal (nNOS), endothelial (eNOS), and inducible (iNOS) NO synthase (NOS), are upregulated in the nervous system under different pathological conditions, including neuropathic pain (Schmidtko et al., 2009). There is strong evidence indicating the specific involvement of iNOS in the onset and progression of neuropathic pain. Among all isoforms, iNOS is most closely associated with inflammation and pain and its expression is increased in dorsal root ganglia and spinal cord in neuropathic pain states (Martucci et al., 2008). SNI mice showed increased spinal levels of iNOS that were abolished by treatment with LEO. Since iNOS inhibitors have been indicated as useful for the treatment of neuropathic pain (De Alba et al., 2006; LaBuda et al., 2006; Ahlawat and Sharma, 2018), this anti-inflammatory effect could, at least in part, explain the antinociceptive effects of LEO.

iNOS is under the transcriptional control of several transcription factors, including nuclear factor-κB (NF-κB) (Kleinert et al., 2004). In the nervous system, NF-κB consists of homo- and heterodimers (p50/p65). Under basal conditions, NF-κB is mainly located within the cytoplasm and is complexed to the inhibitory subunit (IκB), keeping NF-κB in an inactive state. Following specific stimuli, IκB is phosphorylated and degraded. This process releases NF-κB and promotes its translocation from cytosol to the nucleus to modulate the expression of NF-κB-dependent inflammatory genes (Mincheva-Tasheva and Soler, 2013). NF-κB triggers a self-perpetuating process resulting, progressively, in neuropathic pain and many small molecules have been reported to provide protection against neuropathic pain by blocking the NF-κB signaling (Lee et al., 2011; Zhou et al., 2014). However, LEO was unable to counteract the activation of NF-κB, indicating that this pathway is not prominently involved in the mechanism of antihyperalgesic activity of LEO.

New pharmacological treatments for pain are being developed based on molecular interactions with the endocannabinoid system (Woodhams et al., 2017). Although phytocannabinoids from *Cannabis sativa* are ligands of the CB1 and CB2 receptors and are approved for certain pain conditions, the blockade of the endocannabinoids metabolism through FAAH and MAGL inhibition may be a new analgesic approach for neuroinflammatory diseases (Chiurchiù et al., 2018). This is the first time that LEO is reported as a potential FAAH/MAGL inhibitor, which could explain at least in part the analgesic effects in this model of neuropathic pain.

The main components of lavender are linalool, linalyl acetate, ß-ocimene, terpinen-4-ol, 1,8-cineole, and camphor, but the percentage of single constituents varies in different species (Cavanagh and Wilkinson, 2002; Woronuk et al., 2011). In this study, the major components of LEO were linalyl acetate and linalool, accordingly to what described for the composition of lavender oil, obtained from the flowers of *L. angustifolia* (Cavanagh and Wilkinson, 2002).

Essential oils containing linalool and linalyl acetate as major volatile constituents, such as bergamot (*Citrus bergamia*) and yarrow (*Achillea millefolium* L.) essential oils, or purified linalool, showed the capability to reduce ERK and JNK phosphorylation and counteracted the induction of iNOS (Peana et al., 2006; Chou et al., 2013; Kuwahata et al., 2013; Peng et al., 2014), suggesting that these components might be prominently involved in the molecular mechanism of the antihyperalgesic activity of LEO.

Lavender has a long history of medicinal use as antidepressive, anxiolytic, sedative, and calming therapy (Cavanagh and Wilkinson, 2002; Koulivand et al., 2013), properties that have been confirmed by some recent clinical trials (Akhondzadeh et al., 2003; Woelk and Schläfke, 2010; Conrad and Adams, 2012) as well as preclinical works explaining molecular mechanisms of action (López et al., 2017). Neuropathic pain has been associated with a worse quality of life than general population (Doth et al., 2010), largely due to the presence of comorbidities, such as poor sleep, anxiety, and depression, producing a high socioeconomic impact on society (Langley et al., 2013; Torta et al., 2017). We, thus, tested the efficacy of LEO in an anxiety-inducing environment and in a behavioral despair paradigm. Oral administration of LEO promoted an antidepressant-like and anxiolytic-like activity when administered at antihyperalgesic doses. The capability of LEO to positively modulate anxiety and mood simultaneously to the antinociceptive activity would greatly improve the overall symptomatology of neuropathic pain patients with relevant clinical benefit. Treatments for mood disorders are often accompanied by weight gain. Conversely, LEO at active doses reduced food consumption, representing a clinical advantage in case of long-term therapies.

LEO induced its antihyperalgesic effect in the absence of any visible alteration of locomotor behavior or other adverse behavioral effect. These findings show that oral administration of this essential oil can produce a sustained pain relief accompanied by an encouraging tolerability profile. Our positive results are supported by the available literature data. LEO has been granted Generally Recognized as Safe status by the Food and Drug Administration (21CFR182.202015), as indication of its safety when used as a dietary supplement (Food and Drug Administration, 2016). The oral administration of many essential oils in their undiluted form is not recommended due to their irritant, inflammatory, or cytotoxic effects, requiring dilution or even avoidance. LEO is usually used in an undiluted form (topically or orally) and it appears to be well tolerated. Poisoning by lavender is uncommon. In mice, the oral LD50 is 13.5 ± 0.9 g/kg (Jenner et al., 1964). Even though observational studies and long-term trials are necessary to establish the safety of long-term use of LEO for the treatment of neurological disorders, short-term therapy is considered safe (Koulivand et al., 2013), further confirming the encouraging safety profile observed in the present study.

In conclusion, we demonstrated that the oral administration of LEO reduced SNI-induced neuropathic pain symptoms in mice. These effects appeared simultaneously to an antidepressant-like and anxiolytic-like activity, at doses devoid of behavioral side effects. Our data suggest that inhibition of spinal ERK and JNK phosphorylation, and the reduction of iNOS expression by oral LEO may be involved in antineuropathic effects. Oral administration of LEO might represent a therapeutic perspective in the management of neuropathic pain conditions.

## Ethics Statement

The experimental protocol was carried out after approval by the Animal Care and Research Ethics Committee of the University of Florence, Italy, under license from the Italian Department of Health (54/2014-B) and in compliance with international laws and policies (Directive 2010/63/EU of the European parliament and of the council of 22 September 2010 on the protection of animals used for scientific purposes; Guide for the Care and Use of Laboratory Animals, US National Research Council, 2011). All studies involving animals are reported in accordance with the ARRIVE guidelines for experiments involving animals.

## Author Contributions

MS and FL performed the *in vivo* experiments and the data analysis. VL performed the *in vitro* endocannabinoid bioassay, supervised the manuscript, supplied and analyzed the sample of lavender essential oil. NG conceived the study, set up the protocols, and wrote the manuscript.

### Conflict of Interest Statement

Universidad San Jorge has received financial support from Pranarom for research purposes. The funders had no role in study design, data collection, analysis, decision to publish, or preparation of the manuscript.

## Abbreviations

i.p.: Intraperitoneal
FAAH: Fatty acid amide hydrolase
LEO: Lavender essential oil
MAGL: Monoacylglycerol lipase
NORT: Novel object recognition test
p.o.: Per os
SNI: Spared nerve injury
TST: Tail suspension test.
