# Supplementary figures and images for: Lavender (Lavandula angustifolia Mill.) Essential Oil Alleviates Neuropathic Pain in Mice With Spared Nerve Injury
Source: Front Pharmacol. 2019 May 9;10:472. doi: 10.3389/fphar.2019.00472 (PMC6521744; doi:10.3389/fphar.2019.00472)

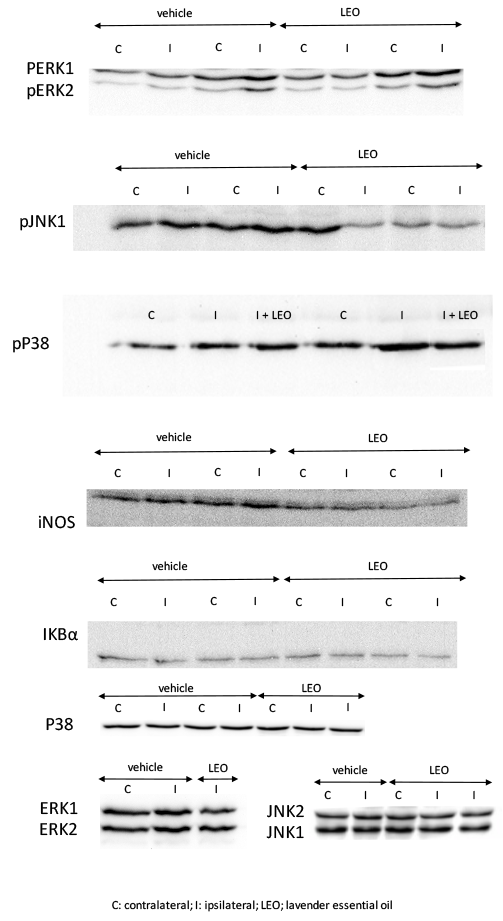

Supplement: Supplementary file 1 [file Image_1.TIF]
